# Effect of a diet enriched with omega-6 and omega-3 fatty acids on the pig liver transcriptome

**DOI:** 10.1186/s12263-016-0517-4

**Published:** 2016-03-17

**Authors:** Agnieszka Szostak, Magdalena Ogłuszka, Marinus F. W. te Pas, Ewa Poławska, Paweł Urbański, Edyta Juszczuk-Kubiak, Tadeusz Blicharski, Chandra Shekhar Pareek, Jenelle R. Dunkelberger, Jarosław O. Horbańczuk, Mariusz Pierzchała

**Affiliations:** 1 Institute of Genetics and Animal Breeding, Polish Academy of Science, Jastrzębiec, Poland; 2 Animal Breeding and Genetics Centre, Wageningen UR Livestock Research, Lelystad, The Netherlands; 3 Functional Genomics Laboratory, Faculty of Biology and Environment Protection, Nicolaus Copernicus University, Toruń, Poland; 4 Department of Animal Science, Iowa State University, Ames, IA USA

**Keywords:** Fatty acid profile, Transcriptome, Omega-3 fatty acids, Omega-6 fatty acids, Liver, Pig

## Abstract

The optimal ratio of omega-6 to omega-3 polyunsaturated fatty acids (PUFAs) is important for keeping the homeostasis of biological processes and metabolism, yet the underlying biological mechanism is poorly understood. The objective of this study was to identify changes in the pig liver transcriptome induced by a diet enriched with omega-6 and omega-3 fatty acids and to characterize the biological mechanisms related to PUFA metabolism.

Polish Landrace pigs (*n* = 12) were fed diet enriched with linoleic acid (LA, omega-6) and α-linolenic acid (ALA, omega-3) or standard diet as a control. The fatty acid profiling was assayed in order to verify how feeding influenced the fatty acid content in the liver, and subsequently next-generation sequencing (NGS) was used to identify differentially expressed genes (DEG) between transcriptomes between dietary groups. The biological mechanisms and pathway interaction networks were identified using DAVID and Cytoscape tools. Fatty acid profile analysis indicated a higher contribution of PUFAs in the liver for LA- and ALA-enriched diet group, particularly for the omega-3 fatty acid family, but not omega-6. Next-generation sequencing identified 3565 DEG, 1484 of which were induced and 2081 were suppressed by PUFA supplementation. A low ratio of omega-6/omega-3 fatty acids resulted in the modulation of fatty acid metabolism pathways and over-representation of genes involved in energy metabolism, signal transduction, and immune response pathways.

In conclusion, a diet enriched with omega-6 and omega-3 fatty acids altered the transcriptomic profile of the pig liver and would influence animal health status.

**Electronic supplementary material:**

The online version of this article (doi:10.1186/s12263-016-0517-4) contains supplementary material, which is available to authorized users.

## Introduction

A balanced ratio of omega-6 to omega-3 polyunsaturated fatty acids (PUFAs) plays an essential role in multiple biological processes and maintains the metabolic homeostasis. It is widely accepted that long-chain PUFAs are considered as a bioactive molecules, which serve beneficial outcomes to health. However, the mechanism responsible for a health-promoting effect as a result of fatty acid consumption is still not fully clarified.

Polyunsaturated fatty acids, particularly omega-6 or omega-3 family, counteract cardiovascular (Stanley et al. 2013) and metabolic disorders (Warensjo et al. 2006). Efficacy of omega-3-rich diet for reducing blood cholesterol levels and decreasing risk of cardiovascular disease, arthritis, neurodegenerative diseases (Lands 2012), and risk of progression to psychotic disorders (Amminger et al. 2015) is unambiguous. Omega-3 PUFAs derivatives can act as signaling molecules, modulating the anti-inflammatory response, and controlling cellular processes involved in programmed cell death (apoptosis), lymphocyte proliferation, suppression of inflammatory cytokines, and phagocytosis (de Pablo and Alvarez de Cienfuegos 2000; Kruger et al. 2010; Wymann and Schneiter 2008). Since omega-6 PUFAs are involved in the regulation of eicosanoid synthesis, they control the activity of the immune system. They are also associated with a reduced risk of cardiovascular diseases. Many recent studies have investigated fish oils and plant oils (for instance linseed and rapeseed oils) rich in omega-3 fatty acids, indicating the protective actions against the development of cardiovascular and metabolic diseases such as obesity, type II diabetes, and metabolic syndrome (Hanke et al. 2013; Lionetti et al. 2014; Morine et al. 2011). Especially, a low omega-6/omega-3 ratio decreases the risk of metabolic diseases (Jump 2011).

In regard to plant oils, diet can provide short-chain linoleic acid (LA) and α-linolenic acid (ALA), which are the main precursors of omega-6 and omega-3 fatty acids, respectively, and are considered as essential fatty acids for humans and other mammals. They can be stored in tissues or converted into long-chain fatty acids such as arachidonic acid (AA), eicosapentaenoic acid (EPA), and docosahexaenoic acid (DHA), which are subsequently involved in the synthesis of hormones such as prostaglandins, prostacyclins, thromboxanes, or leukotrienes (Aro 2003; Harris et al. 2006). There is now significant evidence that one of the mechanisms by which omega-6 and omega-3 fatty acids exert their protective role is interaction with nuclear receptors. They are the natural ligands that influence gene expression and regulate the activity of numerous transcription factors, including sterol regulatory element-binding protein (SREBP), peroxisome proliferator-activated receptor (PPAR), liver X receptor (LXR), farnesoid X receptor (FXR), and retinoid X receptor (RXR) (Getek et al. 2013; Gormaz et al. 2010; Vallim and Salter 2010).

In our studies, we have chosen the pig as an animal model for humans. Such a model has additional advantage because of the relatively similar anatomy, body morphology, physiological and metabolic processes to humans as well as high sequence homology, and a similar chromosomal structure to the human genome, compared to other domestic animal species (Lunney 2007). Therefore, processes revealed in the pig transcriptome are more likely to be similar to those in humans. For these reasons, the pig is considered to be a good animal model for nutrigenomic and metabolic research.

Since the liver is an organ which plays a major role in the regulation of the availability of lipids for other tissues by modulating the uptake and synthesis of lipids and the secretion of lipoproteins, it is adequate to study the fatty acid metabolism dysregulations (Nguyen et al. 2008). Besides adipose tissue, the liver is one of the main locations of synthesis for both cholesterol and triglyceride fatty acids (Bergen and Mersmann 2005). The synthesis of triglycerides depends largely on the availability of fatty acids, which are derived from de novo synthesis, received from diet, or released from tissues (Vallim and Salter 2010). Fatty acid oxidation occurs in hepatic mitochondria and peroxisomes. Dietary fatty acids affect de novo lipogenesis in the liver by controlling the expression of the key enzymes of fatty acid synthesis, particularly acetyl-CoA carboxylase (ACC) and fatty acid synthase (FAS) by PUFA (Guillevic et al. 2009) or stearoyl-CoA desaturase (SCD) by monounsaturated fatty acids (MUFA) (Ntambi 1999).

Nowadays, high-throughput technology, such as next-generation sequencing (NGS), becomes increasingly popular, providing identification of changes in the transcriptome in nutrigenomics investigations (Chen et al. 2011; Li et al. 2011), further validated by standard quantitative real-time PCR (qRT-PCR) (Nygard et al. 2007; Shi and Chiang 2005). The objective of this study was the use of the RNA-Seq approach to identify changes in the pig liver transcriptome induced by a diet enriched with LA (omega-6 family) and ALA (omega-3) fatty acids and to indicate the general biological mechanisms underlying the effects related to PUFA metabolism.

## Materials and methods

### Animals and treatments

**Fig. 1:**
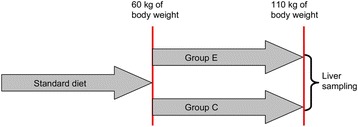
Experimental design. The first stage of feeding was composed of a standard diet until the pigs reached 60 kg in body weight. In the second stage, the pigs were divided into two groups (six pigs per group): the experimental (E) group—fed a diet enriched with LA and ALA, which contained 660 mg and 64 mg/100 g diet, respectively; and the control (C) group—fed a standard diet which contained 268 mg LA/100 g and 25 mg ALA/100 g

The experiment was carried out on Polish Landrace pigs (*n* = 12) housed at a commercial farm (Wronie, Poland) in two separate pens under standard conditions. The first stage of feeding was composed of standard diet appropriate for age group and included all of the pigs, which were fed until reaching 60 kg in body weight (BW) (see scheme in Fig. 1). In the second fattening period, the pigs were divided into two feeding groups (six pigs per group) and fed diets differing in omega-6 and omega-3 fatty acid content based on linseed and rapeseed oil supplementation. In the control group (C, *n* = 6), feed contained 268 mg LA/100 g (linoleic acid—omega-6) and 25 mg ALA/100 g (α-linolenic acid—omega-3 family). The experimental diet (E, *n* = 6) was enriched with LA and ALA and contained 660 mg and 64 mg/100 g diet, respectively. The feeding experiment was performed on an equal number of pigs from each treatment group, and all experiment included gilts. Diets were provided ad libitum with a constant access to water. The fodder mixtures were isoenergetic (13 MJ EM per kg of mixture) and isoprotein balanced (15.5 % crude protein), while differing in fat content ranging from 1.78 % (C group) to 4.72 % (E group). Diets were composed of the same components, except linseed and rapeseed oils, which were provided as a polyunsaturated fatty acid source (Table 1). The feed was administered for approximately 4 months until slaughter at the average BW of 110 kg. All animals were characterized by aligned age and body weight. Pigs were sacrificed by electrical stunning and exsanguination, according to industry standards. After slaughter, liver samples were excised and immediately frozen in liquid nitrogen and further stored at −80 °C. Slaughter procedure was carried out with the required permits and according to Minister of Agriculture and Rural Development dated April 2, 2004.

**Table 1:** Raw material composition of mixtures (%)

Ingredients	Diets	
	C	E
Rapeseed meal	6	7.2
Soybean meal	9	9.2
Wheat	54	49.6
Barley	28.5	28.5
Linseed oil	–	2
Rapeseed oil	–	1
Other	2.5	2.5
Total	100	100

### Fatty acid profiling

The fatty acid composition in the liver was assayed in order to verify how feeding influenced the fatty acid content in tissues. Subsequently, the ratio of omega-6/omega-3 fatty acids in the liver was calculated for both dietary groups and used as the basis for further investigation. For *p*-value determination, unpaired Student’s test was used.

The fatty acid profile analysis was performed using the chloroform-methanol (2:1 *v*/*v*) method (Folch et al. 1957) on 1 g of liver sample. Fatty acid methyl esters were determined by gas chromatography (GC-FID) on the Agilent 7890 Series GC system equipment with a Hewlett-Packard-88 capillary column (60 m × 0.25 mm × 0.20 μm) (Agilent, J&W GC Columns, USA) with helium as a carrier gas. The details of the analysis are described in Polawska et al. (2013). Fatty acids were identified by comparison with fatty acid standards (Supelco 37 Component FAME mix, 47885-U—10 mg/ml in methylene chloride, analytical standard, Sigma-Aldrich Co.) and expressed in g/100 g fatty acid methyl esters (FAME).

### Gene expression analysis

#### Next-generation sequencing

The total RNA for sequencing were purified from 20 mg of tissue sample of 12 pigs (6 animals in control group and 6 animals in PUFA treatment group) using the messenger RNA (mRNA) extraction kit RNeasy Fibrous Tissue Mini Kit (Qiagen, Hilden, Germany) following the manufacturer’s recommendations. The concentration and purity of total RNA were checked using the UV/Vis spectrophotometer (Actgene, New Jersey, USA) at 260 nm, and the integrity was evaluated using a microfluidic assay on Bioanalyzer (Agilent Technologies, Inc., Santa Clara, CA, USA). Only high quality RNA extracts (RNA integrity number (RIN) ≥8) were used for pooling within each treatment group using equal amounts of RNA per animal.

Next-generation sequencing data were obtained from pooled RNA samples within each group to ensure the most robust transcriptome. Construction of complementary DNA (cDNA) libraries for RNA sequencing was prepared using the TruSeq RNA Sample Prep Kit v2 (Illumina, San Diego, CA, USA). RNA-Seq analysis was performed for identification of transcriptional changes using MiSeq instrument (Illumina) according to the manufacturer’s recommendations at the laboratory of Genomed (http://www.genomed.pl/) using paired-end libraries. Two replicates of each pool independent for library synthesis and sequencing were done. The number of reads equivalent to mapped reads (reads per kilobase per million (RPKM)) was used to normalize the expression of each gene. Benjamini and Hochberg test (an error rate 0.05) and false discovery rate correction test (FDR < 0.05) were used for adjusting the *p*-value. In this study, a minimum twofold change (FC ≥ 2) difference in gene expression between treatment groups was used as the inclusion criteria for a gene. Reads were mapped to the *Sus scrofa* v. 10.2 genome as a reference genome. The quality of obtained data was checked in regard to the presence and abundance of contaminating sequences, average read length, and GC content. The NGS experiment conformed to the MIAME guidelines (Brazma et al. 2001) except for the microarray design. The RNA-Seq raw data were deposited in NCBI GEO (Gene Expression Omnibus) (Barrett et al. 2013) under accession number GSE72123.

#### Quantitative real-time PCR

Total cellular RNA was extracted according to the Chomczyński protocol (Chomczynski and Sacchi 1987; Chomczynski and Sacchi 2006). The samples of liver tissue (20 mg) were lysed with TRIzol reagent (Invitrogen, Carlsbad, CA, USA) and homogenized on MagNA Lyser (Roche Diagnostics, Mannheim, Germany). The concentration and purity of the RNA extracts were measured using UVS-99 micro-volume UV/Vis spectrophotometer (ACTgene, Piscataway, NJ, USA) at 260 nm. Integrity was checked using the Agilent 2100 Bioanalyzer (Agilent Technologies Inc., Santa Clara, CA, USA) based on the RNA integrity number (RIN) parameter. The RIN values for all samples were higher than 7.5. Genomic DNA was removed from RNA extracts by incubation with RQ1 RNase-free DNase (Promega, Madison, WI, USA) for 15 min at 25 °C. The RNA extracts were stored in RNase-free water at −80 °C. The reverse transcription reaction was performed using the Transcriptor First Strand cDNA Synthesis Kit (Roche Applied Science, Penzberg, Germany) from 1.5 μg of total RNA in a final volume of 20 μl using the oligo-dT primers.

Quantitative real-time PCR was performed on 30 individual samples (biological replicates—15 samples for each dietary group) to validate the reliability of RNA-Seq gene expression data. For confirmation, we have chosen the differentially expressed genes EXTL1, APOA4, APOA5, ACSL1, ELOVL6, FASN, COL1A1, DGAT2, FADS1, FADS2, and MMP2. Most of the selected genes are involved in lipid metabolism. Gene expression was normalized by the use of topoisomerase (DNA) II beta (TOP2B) (Nygard et al. 2007; Pierzchala et al. 2011) as a most stable gene revealed by NormFinder algorithm, which was used to determine the optimal normalization gene among a set of candidates (GAPDH, TBP, TOP2B). The primer sets for real-time PCR were designed using Primer-BLAST tool (Ye et al. 2012) (www.ncbi.nlm.nih.gov/tools/primer-blast/) and Primer Premier 5.0 software (PREMIER Biosoft, Palo Alto, CA, USA). The sequences of the primers and their annealing temperatures are described in Table 2.

**Table 2:** Oligonucleotide primer sequences used for quantitative real-time PCR experiments

Gene	Sequences (5′–3′)	Amplicon size (bp)	Annealing temperature (°C)	GenBank accession no.
DGAT2	For. GACCCTCATAGCTGCCTACTC	136	59	NM_001160080.1
	Rev. CAGCACGGAGATGACCTGTA			
ELOVL6	For. GAACACGTAGCGACTCCGAA	178	59	XM_003357048.2
	Rev. ATGCCGACCGCCAAAGATAA			
EXTL1	For. TGGGATGGGCACTGTGAGC	78	59	XM_003356212.1
	Rev. GCAGAAGGTGGCATTGGGTA			
MMP2	For. CGGACAAAGAGTTGGCTGTG	158	59	NM_214192
	Rev. CATGGTCTCGATGGTGCTCT			
COL1A1	For. TTCAGCTTTGTGGACCTCCG	136	59	XM_005668927
	Rev. CGTTCTGTACGCAGGTGACT			
ACSL1	For. TCAGAAGGTTGCCAGTGAAG	115	59	NM_001167629
	Rev. CTGGAGGAGAGGATCAGAGAATA			
APOA4	For. CAGCAGCTCAACACTCTCTT	142	59	NM_214388
	Rev. GAATCTCCTCCTTCAGCTTCTC			
APOA5	For. GGAAGAGAAGGGAAGAAGGAAG	83	59	NM_001159308
	Rev. CATCGGCCAACAGGGATATT			
FADS1	For. GGGCCTTGTGAGGAAGTATATG	104	59	NM_001113041
	Rev. GGAACTCATCTGTCAGCTCTTT			
FADS2	For. CCTTACAACCACCAGCATGA	118	59	NM_001171750
	Rev. CCAAGTCCACCCAGTCTTTAC			
FASN	For. CTTGTCCTGGGAAGAGTGTAAG	83	59	NM_001099930
	Rev. AGATGGTCACCGTGTCTTTG			
TOP2B	For. AACTGGATGATGCTAATGATGCT	137	59	NM_001258386.1
	Rev. TGGAAAAACTCCGTATCTGTCTC			

The reaction mixture for real-time PCR consisted of 4.5 μl of cDNA template (1/20 dilution), 5 pmol reaction primers, and 5 μl SYBR Green I in a final volume of 10 μl. All PCR reactions were performed in duplicate. For each gene, negative controls without the cDNA template were included. The reactions were performed in a 96-well optical plate on the Light Cycler 96 instrument (Roche Applied Science, Germany). Cycling conditions were as follows: pre-incubation at 95 °C for 5 min followed by 45 amplification cycles of 95 °C (10 s), 59 °C (10 s), and 72 °C (10 s). After each experiment, the qRT-PCR products were verified for specific amplification by melting curve analysis: 95 °C (5 s), 70 °C (1 min), heating to 95 °C, and cooling step to 37 °C for 30 s. Additionally, PCR product quality and specificity with regard to the applied primer pairs were checked using gel electrophoresis.

The gene expression levels were calculated using the LinRegPCR analysis program according to the baseline estimation method (Ramakers et al. 2003; Ruijter et al. 2013; Ruijter et al. 2009). In order to evaluate the relative gene expressions for independent samples, bilateral Student’s test was used and *p*-value <0.05 was considered significant. The quantitative real-time PCR was performed according to the MIQE guidelines (Bustin et al. 2013).

#### Bioinformatics analysis

For transcriptome assembling and dispersion evaluation, the CLC Genomics workbench v. 6.0. was used. Since pooled samples were used in the NGS experiment, the expression variance for each gene was estimated by using the variance across the four sequencing for that gene. This “blind” method of dispersion estimate is used in the absence of biological replicates, even though it can overly conservative statistical significance testing of differential gene expression (Anders and Huber 2010).

All data processing steps were performed using Lasergene Genomic Suite v. 5 software package (DNASTAR, Madison, WI, USA) for differential gene expression evaluation. The first step included selection of the genes that showed high variation for all identified gene transcripts. Only genes that demonstrated more than a twofold change in differential expression between dietary groups with a *p*-value ≤0.05 for the *T* test were selected for further analysis. Additionally, genes that demonstrated dietary group-specific expression (and remained below the detection level in the opposite dietary group) were investigated separately.

Biological mechanisms underlying DEGs were investigated using the DAVID v. 6.7 software (the Database for Annotation, Visualization, and Integrated Discovery) (http://david.abcc.ncifcrf.gov/) (Huang et al. 2009a; Huang et al. 2009b). The sets of genes were uploaded using ENTREZ gene IDs. To obtain maximal annotation results, the pig gene IDs were converted to the homologues human gene IDs. The *p*-values of the enrichment of the number of genes in biological mechanisms were evaluated by Benjamini correction, and values less than 0.05 were considered significant. The data were analyzed for the following situations: (1) all DEGs, (2) DEGs with expression levels below the detection level in the C group, and (3) DEGs with expression levels below the detection level in the E group. The analysis performed using the DAVID software allowed to derive the comprehensive information about the genes’ participation in biological pathways from the Kyoto Encyclopedia of Genes and Genomes (KEGG) database (http://www.genome.jp/kegg/) (Kanehisa and Goto 2000; Kanehisa et al. 2014). The *p*-values calculated in the DAVID software indicate the importance of the general biological mechanisms revealed in all animals but do not distinguish the inter-individual differences. For this purpose, we examined the alterations in the pig transcriptome in pooled liver samples per treatment group in order to clear out the bias caused by variation in a heterogeneous group of individuals derived from the general population. Apart from RNA-Seq, qRT-PCR was performed for individual samples, and indeed, high variation between individual animals was observed. The physiological effects of dietary omega-6 and omega-3 fatty acid intake were measured in the individual animals which are the consequence of the interactions between the animals’ genotype and the omega-6/omega-3 ratio. Therefore, it was expected that animal-specific mechanisms were observed, which were not intended for our study. In a pooled sample experiment, the contribution of these individual-specific reactions is diluted, while the general mechanisms active in all animals remain emphasized.

Finally, the molecular interaction networks of DEGs were investigated using the Cytoscape v. 3.1.0 software (http://www.cytoscape.org/) (Saito et al. 2012) as a complementary and more comprehensive approach for the identification of central hub genes. The networks of gene relationships were based on the *Homo sapiens* gene annotation databases. Results were visualized using the ClueGO v. 2.1.1. app. of the Cytoscape software to create clusters of functionally related genes, using the gene ontology databases: KEGG, Wiki Pathways, REACTOME, GO Immune System Process, GO Molecular Function, GO Cellular Component, and GO Biological Process.

The probability value was calculated using Enrichment/Depletion (a two-sided hypergeometric test) with Bonferroni correction. The networks of genes were created for the following situations: (1) all DEGs, (2) DEGs expressed only in the E group and were below the detection level for the C group, (3) DEGs expressed only in the C group and were below the detection level in the E group, (4) DEGs expressed only in the C or E group when combined in one network. The parameters “number of genes per node” and “minimum percentage of genes per node” were chosen empirically for different analyses.

## Results

### Animals and fatty acid profiles

There were no significant differences in growth performance and carcass characteristics between the pigs in the two treatment groups (data not shown). However, a tendency toward a lower daily gain and a lower backfat thickness in the experimental group was observed, but it was statistically insignificant. No interaction was observed between addition of PUFA and slaughter weight.

The analysis of the fatty acid profile showed a higher level of PUFAs in the liver of the experimental group (*p*-value = 0.0026), as a consequence of the increased content of PUFAs in the diet. Supplementation of the diet with PUFAs was associated with a higher content of most omega-3 fatty acids in the liver and unchanged or lower omega-6 fatty acid content, hence, pronounced reduction of the omega-6/omega-3 fatty acid ratio (Table 3). Particularly, contents of eicosapentaenoic acid (EPA) (*p*-value = 0.0006) and docosahexaenoic acid (DHA) (*p*-value = 0.0006) of the omega-3 family were higher, while that of arachidonic acid (AA)—omega-6 was lower in the E group in comparison to the C group (*p*-value = 0.0055). Interestingly, the mean value of the omega-6/omega-3 fatty acid ratio was approximately 30 in standard diet group (C group) and approximately 4 for the group with increased content of PUFAs.

**Table 3:** Fatty acid content (g/100 g FAME) in liver samples from two dietary groups. Data are presented as averages

Fatty acids		C diet group		E diet group		*p*-value (*T* test)
		Mean	SD	Mean	SD	
∑ SFA		43.18	9.12	42.35	5.09	0.7041
	C12:0	3.21	2.44	2.31	1.90	0.1754
	C14:0	0.62	0.28	0.58	0.27	0.6893
	C16:0	17.93	4.61	6.17	2.48	0.1114
	C18:0	21.77	5.8	23.42	4.64	0.2824
∑ MUFA		22.96	7.2	19.65	5.38	0.0792
	C16:1 n-7	3.47	4.62	1.85	0.75	0.1094
	C18:1 n-9	17.56	5.42	16.30	4.67	0.3918
	C18:1 n-7	1.93	0.42	1.64	0.23	0.0058**
∑ PUFA		27.23	8.05	33.9	6.28	0.0026*
	C18:2 n-6 (LA)	13.7	3.4	15.61	2.27	0.0284*
	C18:3 n-3 (ALA)	0.82	1.17	3.32	1.17	0.0000**
	C20:3 n-6	3.02	6.09	1.57	2.61	0.5676
	C20:4 n-6 (AA)	12.66	4.18	9.41	2.87	0.0055**
	C20:5 n-3 (EPA)	0.87	0.74	3.16	0.92	0.0006**
	C22:6 n-3 (DHA)	1.88	1.08	3.38	1.02	0.0006**
	∑ Omega-6^a^	25.63	7.72	25.69	4.13	0.9763
	∑ Omega-3^b^	2.16	1.83	8.22	3.50	10^−9^**
Omega-6/omega-3 ratio		32.73	62.02	4.25	3.93	0.0769
Other^c^		6.63		4.1		

*SFA* saturated fatty acids, *MUFA* monounsaturated fatty acids, *PUFA* polyunsaturated fatty acids

^a^LA + ETA + AA

^b^ALA + EPA + DHA

^c^FA not identified according to the used standard

*P < 0.05

**P < 0.01

We did not observe any statistically significant differences in regard to the content of saturated fatty acids (SFA). We observed a lower content of MUFA in the liver of the E group compared to the C group (*p*-value = 0.07).

### Next-generation sequencing

Next-generation sequencing using RNA-Seq approach was performed to characterize the pig liver transcriptome. The number of reads per sample obtained from the NGS was 13,509,248 sequences using paired-end libraries (PE). Among these, 9,812,246 (72.63 %) reads were mapped in pairs, 381,668 (2.83 %) were mapped in broken pairs, and 3,315,334 (24.54 %) reads were not mapped. The average read was 151 base-pair lengths.

In regard to the fragment counting, the total number of fragments was 6,754,624, in which 1,848,501 (27.37 %) remained uncounted. Among 4,906,123 counted fragments (72.63 % of total fragments), 4,401,376 (65.16 %) were unique and 504,747 (7.47 %) were non-specific. An intact pair was counted as one, and broken pairs were ignored.

In total, 20,325 transcripts were annotated to genes revealed by the RNA-Seq technology for the pig liver samples (Table 4). Analysis identified 3565 DEGs between the two dietary groups, 1484 of which were up-regulated and 2081 of which were down-regulated in the E group compared to the C group. The differentially expressed genes identified in the both dietary groups included 2098 annotated transcripts. Results also indicated that expression of certain genes in the liver were unique to a particular dietary group. For instance, 809 genes were unique to the E group while the expression of these genes in the opposite group was below our detection level and 658 genes were unique to the C group.

**Table 4:** Summary of RNA sequencing analysis data of pig liver tissues fed a control (C) diet or the experimental (E) diet consisting of additional omega-6 and omega-3 fatty acids

	Number of DEGs	
Total number of DEGs	3565	
Genes up-regulated among all DEGs	1484 UP^a^	
Genes down-regulated among all DEGs	2081 DOWN^b^	
Expression in both groups	2098	
Highly expressed genes common to both groups	27	
	C diet	E diet
Expression in one group only	658	809
Highly expressed genes (RPKM ≥1000)	41	30

*DEGs* differentially expressed genes

^a^Up-regulated genes—the expression level higher in E diet group than in C diet group

^b^Down-regulated genes—the expression level lower in E diet group than in C diet group

The mean value of the absolute expression level was 67.95 for all reads in both dietary groups. However, high-throughput sequencing results also identified genes that were characterized by an extreme high absolute value of expression level (RPKM ≥1000). Among the 3565 DEGs, 30 genes in the E group and 41 genes in the C group had an extreme high absolute value of expression, 27 of which were common to both dietary groups.

Fold change (FC) for highly expressed genes (RPKM ≥1000) ranged mainly from 2.0 to 5.0. Two genes had higher FC—retinol saturase (RETSAT, FC = 9.1) and serine dehydratase (SDS, FC = 27.3). Among the up-regulated genes characterized by the highest value of absolute expression were as follows: APOA4, APOA5, HMGCS2, ACSL1, and CYP2C49, while highly expressed down-regulated genes were COX1, SULT2A1, HPD, FMO1, and CPS. These genes are mainly related to fatty acid activation, fatty acid β-oxidation, lipid binding, and lipid transport activity (up-regulated) or were enzymes involved in prostaglandin biosynthesis or peroxidase activity (down-regulated).

The mean FC value of all DEGs was 9.4. The next-generation sequencing analysis also revealed genes characterized by a high FC value caused by a low level of expression for genes within one dietary group.

### Quantitative real-time PCR

**Fig. 2:**
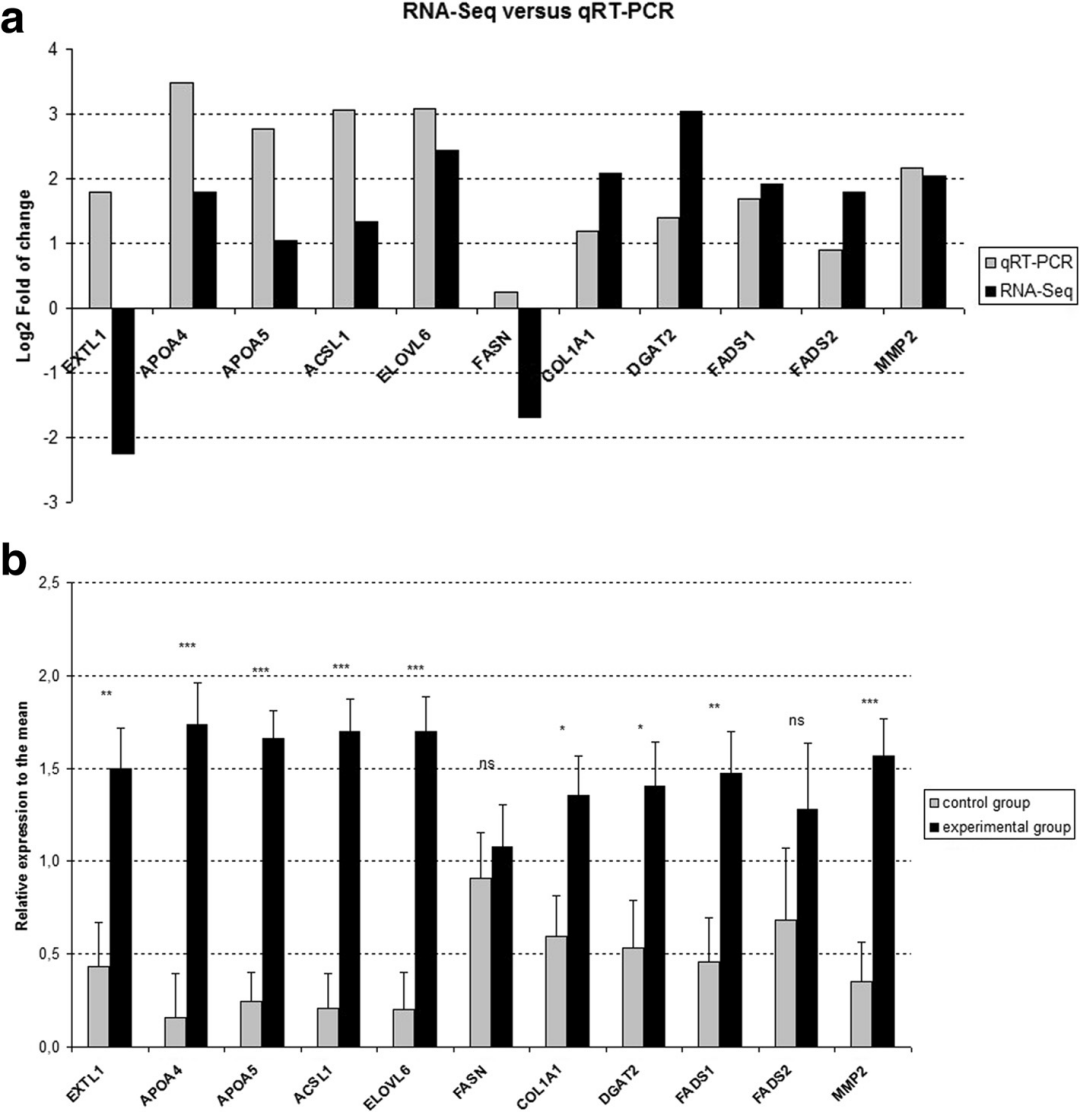
Validation of differentially expressed genes (DEGs) by qRT-PCR. **a** comparison of the RNA-Seq (6 animals per group) and qRT-PCR (15 animals per group), Log2-fold of change between experimental and control groups, **b** individual variability of validated differentially expressed genes in qRT-PCR. For qRT-PCR, fold change was assessed on the basis of mean values of gene expression for 2 dietary groups with 15 individual samples per group. All Ct values were normalized to the topoisomerase (DNA) II beta (TOP2B) gene. Error bars represent standard error of variability among individual samples. *p-value <0,05; **p-value <0,01; ***p-value <0,001, *ns* not significant

Differentially expressed genes selected from RNA-Seq generated data were validated using quantitative real-time PCR. Almost all genes chosen for validation were regulated in the same direction in both approaches excluding EXTL1 and FASN. The differences in Log2-fold of change between both methods were observed as shown in Fig. 2a. The differences in mean relative expression between control and experimental groups were observed (Fig 2b) in agreement with RNA-Seq data using RPKM expression values for almost all genes excluding EXTL1 (opposite results) FASN and FADS2 (statistically insignificant). High variability between the particular individuals among groups was visible.

### Functional categorization of DEGs and pathway analysis

#### Fatty acid metabolism

**Fig. 3:**
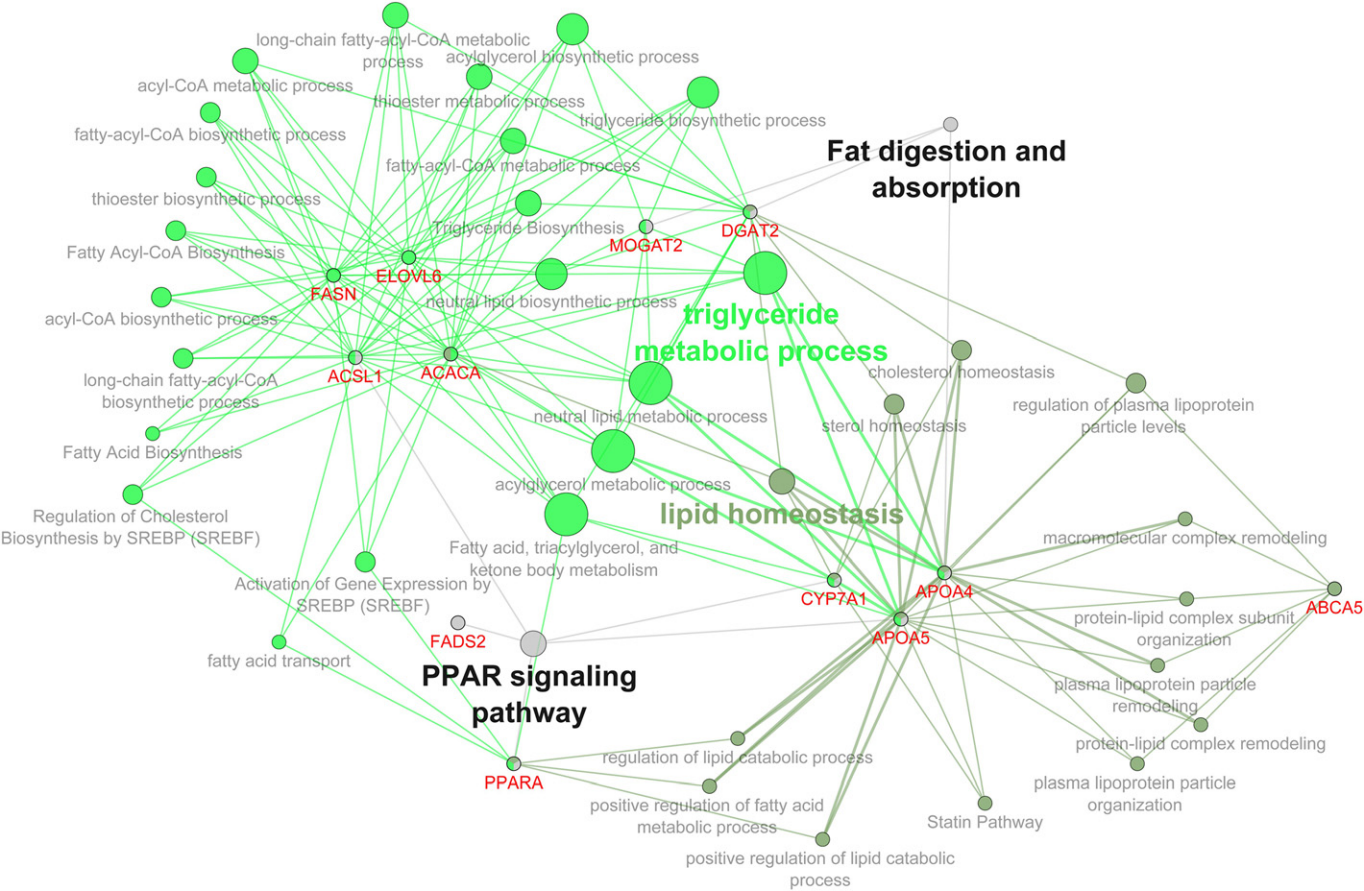
The gene network pathways related to fatty acid metabolism. Visualization generated by Cytoscape-ClueGo software after selection criteria: at least three genes per one pathway node and 4 % of the associated genes from all genes in one node

The different ratio of omega-6/omega-3 fatty acids in the liver was reflected in altered fatty acid metabolism pathways revealed by the Cytoscape-ClueGo software. The visualization of potential networks for selected genes was consistent with previous observations of the most highly expressed genes and revealed the major biological mechanisms related to fatty acid metabolism: triglyceride metabolic process, lipid homeostasis, the PPAR signaling pathway, and fat digestion and absorption (Fig. 3 and Additional file 1).

#### All DEG

To further investigate the biological importance of DEG, we performed gene category analysis in DAVID software. Among gene sets comprising all DEGs, 1623 records were assigned to gene identifiers recognized by the DAVID tool. Table 5 shows 12 significant functional annotation clusters (for more details see Additional file 2). The created clusters represented genes involved in cell membrane-related processes and signal transduction. The cluster with the highest enrichment score (8.6) included 363 genes related to the plasma membrane. The functional clusters that demonstrated the highest number of involved genes were transmembrane proteins in the cellular component category, signal peptide and glycoproteins in the biological processes category, and ion binding for the molecular function category. Taken together, major functions within this group appeared to be related to plasma membrane proteins, extracellular matrix, and cell transport and signaling. Functional classification in the DAVID tool adjusted DEG to terms in the KEGG database and showed the importance of 3 KEGG pathways—cytokine-cytokine receptor interactions (42 genes), focal adhesion (34 genes), and dilated cardiomyopathy (21 genes).

**Table 5:** Functional clusters created for all DEGs

Functional cluster analysis			
Biological function	Enrichment score	Number of involved genes	Ontology
Plasma membrane	8.6	363	CC
Glycoprotein/signal peptide	7.6	487	CC/BP
Transmembrane protein	6.4	646	CC
Extracellular matrix	4.5	115	CC
Ossification	4.0	44	BP
Fibronectin type 3	3.9	36	MF
Protein kinases	3.7	98	BP
Calmodulin binding	3.6	24	MF
Angiogenesis	3.4	39	BP
Ion binding	3.2	414	MF
Cell motion	3.0	63	BP
Cardio-related	3.0	26	BP

Clustering was performed using DAVID software

*CC* cellular component, *BP* biological process, *MF* molecular function

**Fig. 4:**
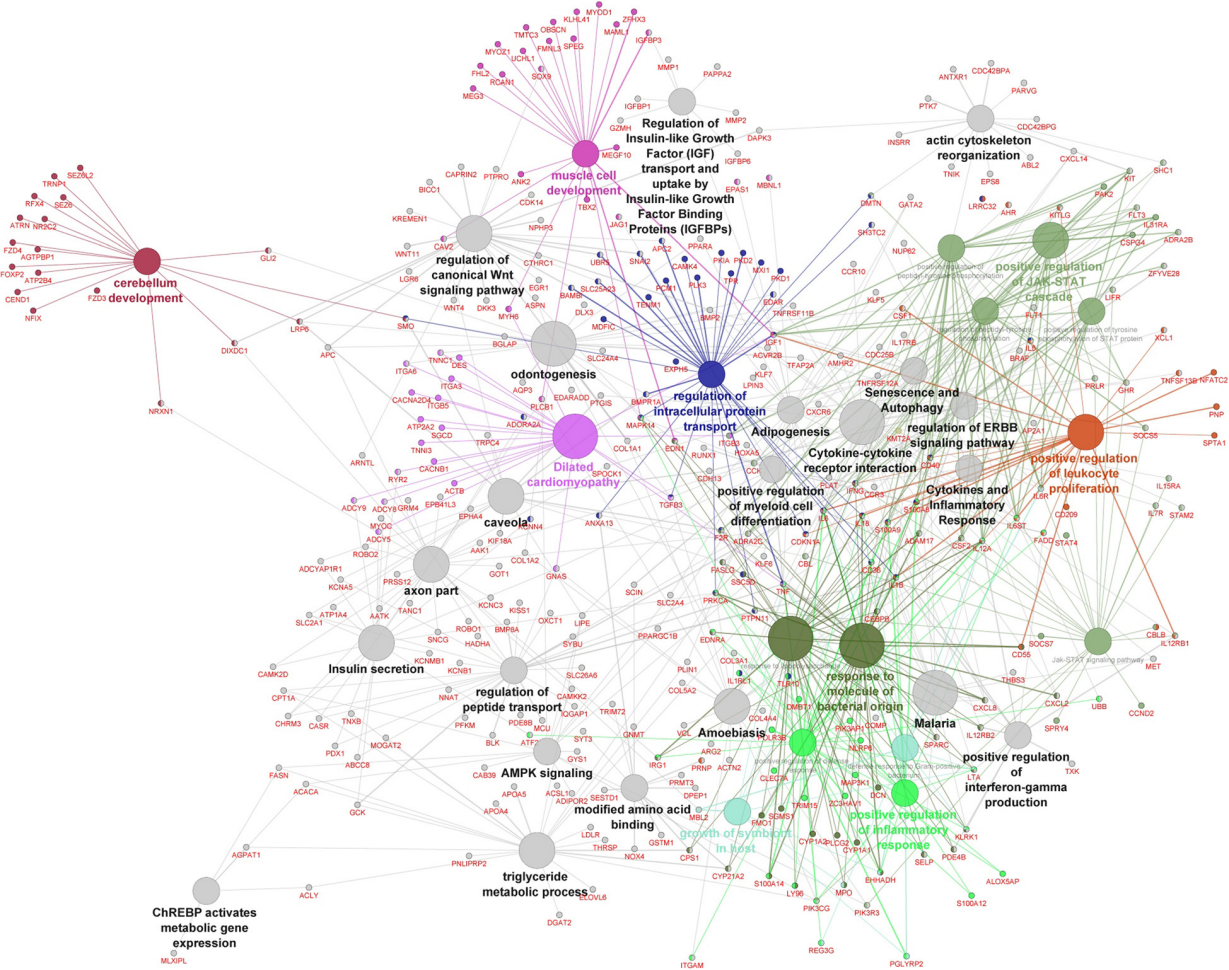
The gene network and biological processes for all DEGs. Figure generated by Cytoscape-ClueGo. The *red* and *green* dots represent the up-regulated and down-regulated genes in the E group compared to the C group, respectively. The *lines* represent interactions among genes. The processes marked in *grey* are processes without direct connections to any of the other processes. The selection criteria for this network were to have at least two genes per node with a minimum of 5 % of the associated genes from all loaded genes in one node. The group percentage was 50 % with a kappa score threshold of 0.4

**Fig. 5:**
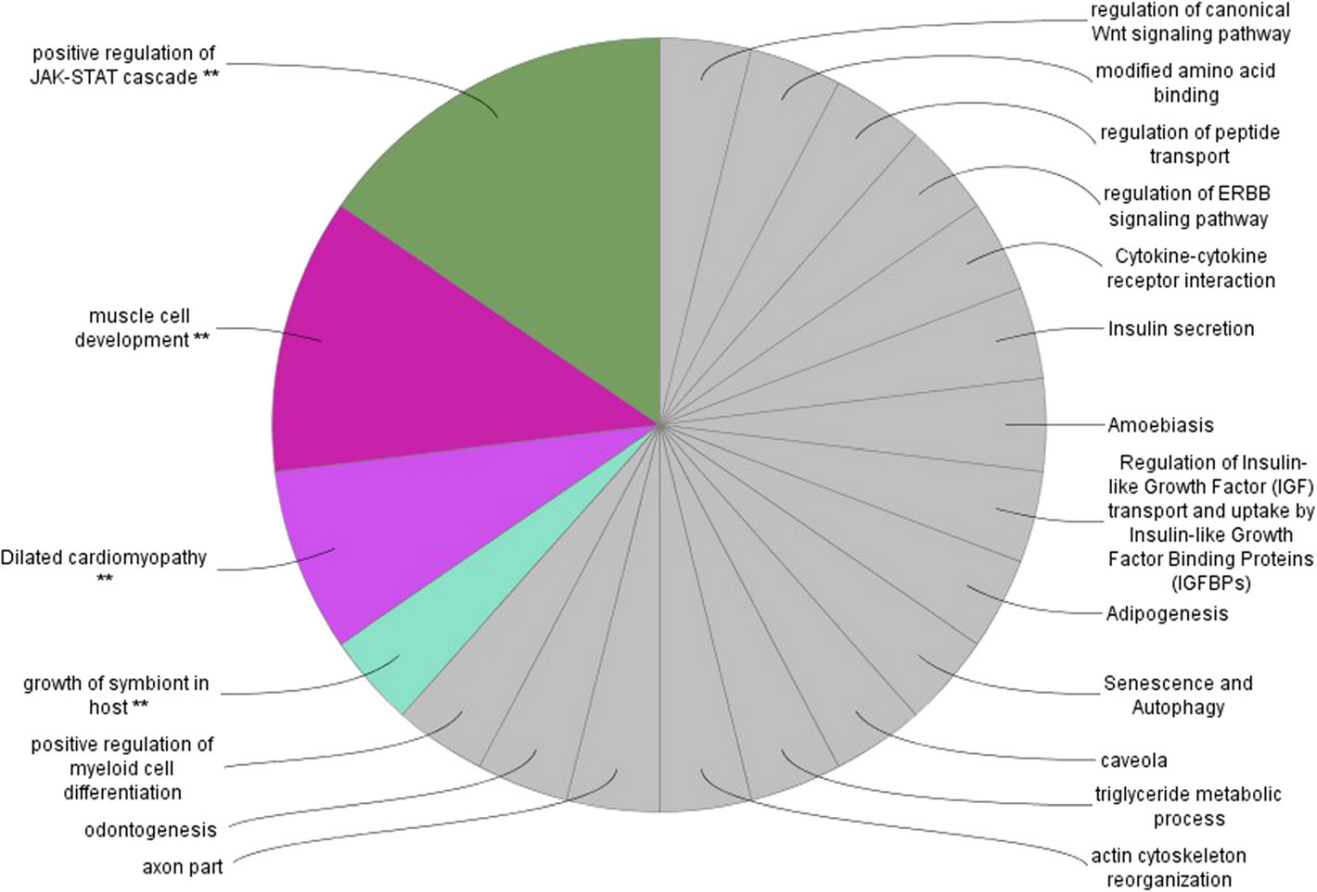
The pathway distribution of the specific clusters for all DEGs. Analysis performed in Cytoscape-ClueGo. The selection criteria for this network were to have at least two genes per node with a minimum of 5 % of the associated genes from all loaded genes in one node. The group percentage was 50 % with a kappa score threshold of 0.4

Cytoscape bioinformatics analysis of potential interactions between proteins for all DEGs was performed on 729 genes (Additional file 3). The major biological functions revealed by Cytoscape-ClueGo software were as follows: positive regulation of the JAK-STAT cascade, muscle cell development, dilated cardiomyopathy, growth of symbiont in the host, positive regulation of leukocyte proliferation, regulation of intracellular protein transport, positive regulation of inflammatory response, response to molecules of bacterial origin, and cerebellum development (Fig. 4). Several of these pathways were grouped around biological themes, such as inflammatory/pathogen response (10 pathways), signaling pathways (4), brain/nerve development (3), carbohydrate/lipid metabolism (3), and muscle development and function (2). The JAK-STAT pathways (on the right, near the immune response pathways) and the pathogen response pathway (in the lower right) were grouped relatively close to the inflammatory response pathways (Fig. 4). Muscle development was located near the top of the figure, above and close to the cardio-related pathways. The main groups representing the brain development pathways are in the upper left, separated from the other pathways. The carbohydrate/lipid metabolism pathway is on the bottom left. The regulation of the intracellular protein transport pathway is located in the center. All signaling-related pathways were dispersed throughout the network. Overall, the processes, which were grouped together, were more related to each other than they were to the others. The distribution of pathways for all DEGs as specific clusters is presented in Fig. 5.

#### Genes expressed only in the experimental group

The analysis in DAVID, which comprised set of genes specific to the E group, recognized and analyzed 359 gene IDs. The results for this subset of genes are presented in Table 6 (more details are listed in the Additional file 4). The cluster with the highest enrichment score was the glycoprotein/transmembrane cluster. The signal peptide and ion transport clusters belong to the functional mechanisms in this category. No pathways were revealed from the KEGG database for the set of genes expressed exclusively in the E dietary group.

**Table 6:** Functional clusters for genes expressed only in E diet group

Functional cluster analysis			
Biological function	Enrichment score	Number of involved genes	Ontology
Glycoprotein/transmembrane	5.4	166	CC
Signal peptide	5.2	129	BP
Plasma membrane	3.8	98	CC
Fibronectin type 3	2.3	13	MF
Ion transport	2.3	41	MF

*CC* cellular component, *BP* biological process, *MF* molecular function

**Fig. 6:**
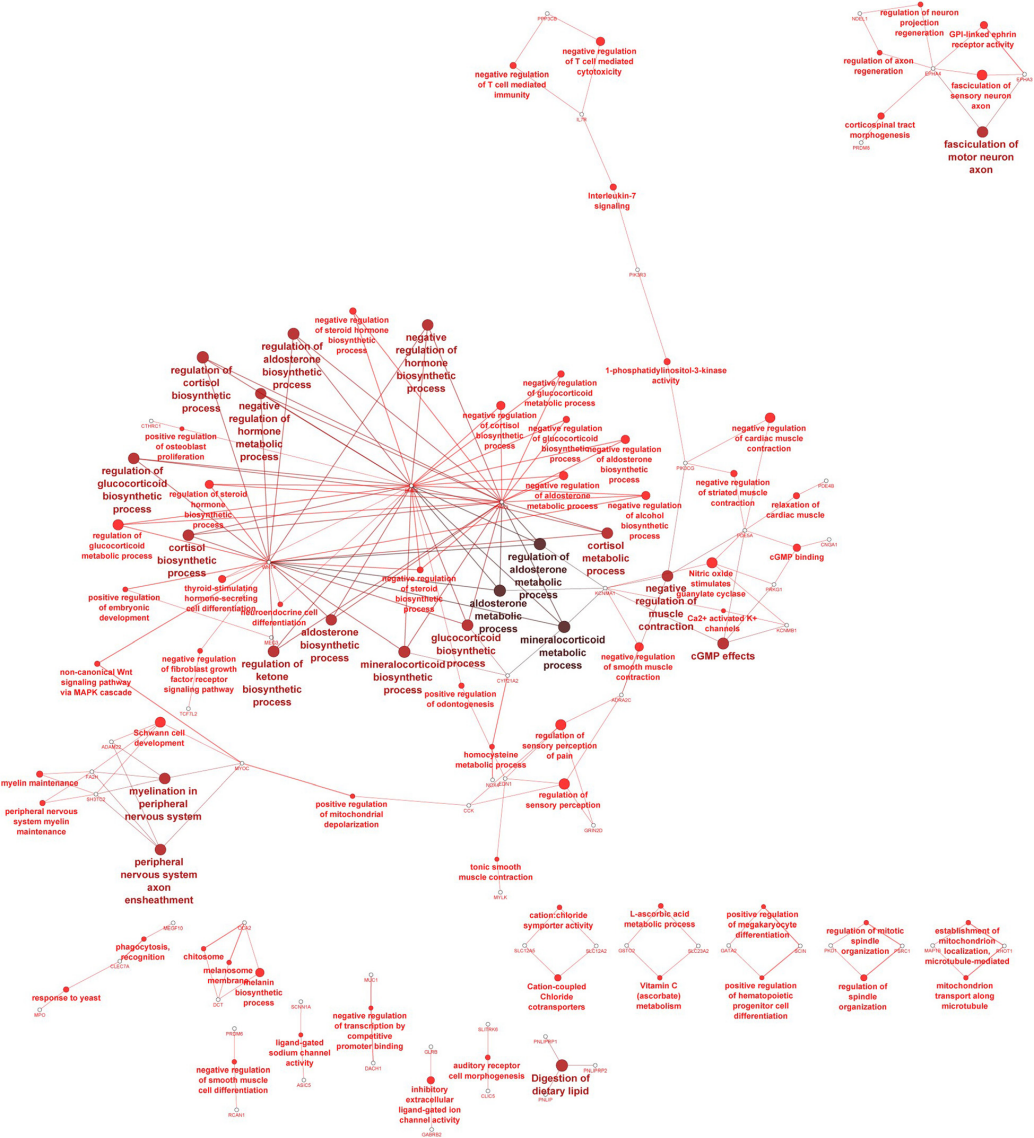
The gene network and biological processes for genes specific to experimental (E) group. Analysis performed in Cytoscape-ClueGo. The selection criteria for this network were to have at least two genes per node with 15 % of the associated genes from all loaded genes in one node. The kappa score threshold was 0.51

Visualization of the molecular interaction networks in Cytoscape for DEGs detected only in the E group was performed for 59 genes (Additional file 5). Selected genes were involved mainly in the regulation of steroid hormone biosynthesis (Fig. 6). Other affected pathways involved muscle contraction, nervous system, signaling, immune response, and ion channels. The results also included a number of small networks that were separated from the main network.

#### Genes expressed only in the control group

In total, 275 DEGs were converted to the DAVID-recognized gene IDs, but no functional cluster was assigned in this group (Additional file 6). The KEGG pathway analysis in DAVID software revealed two molecular interaction networks: cytokine-cytokine receptor interaction (12 genes) and type I diabetes mellitus (5 genes).

**Fig. 7:**
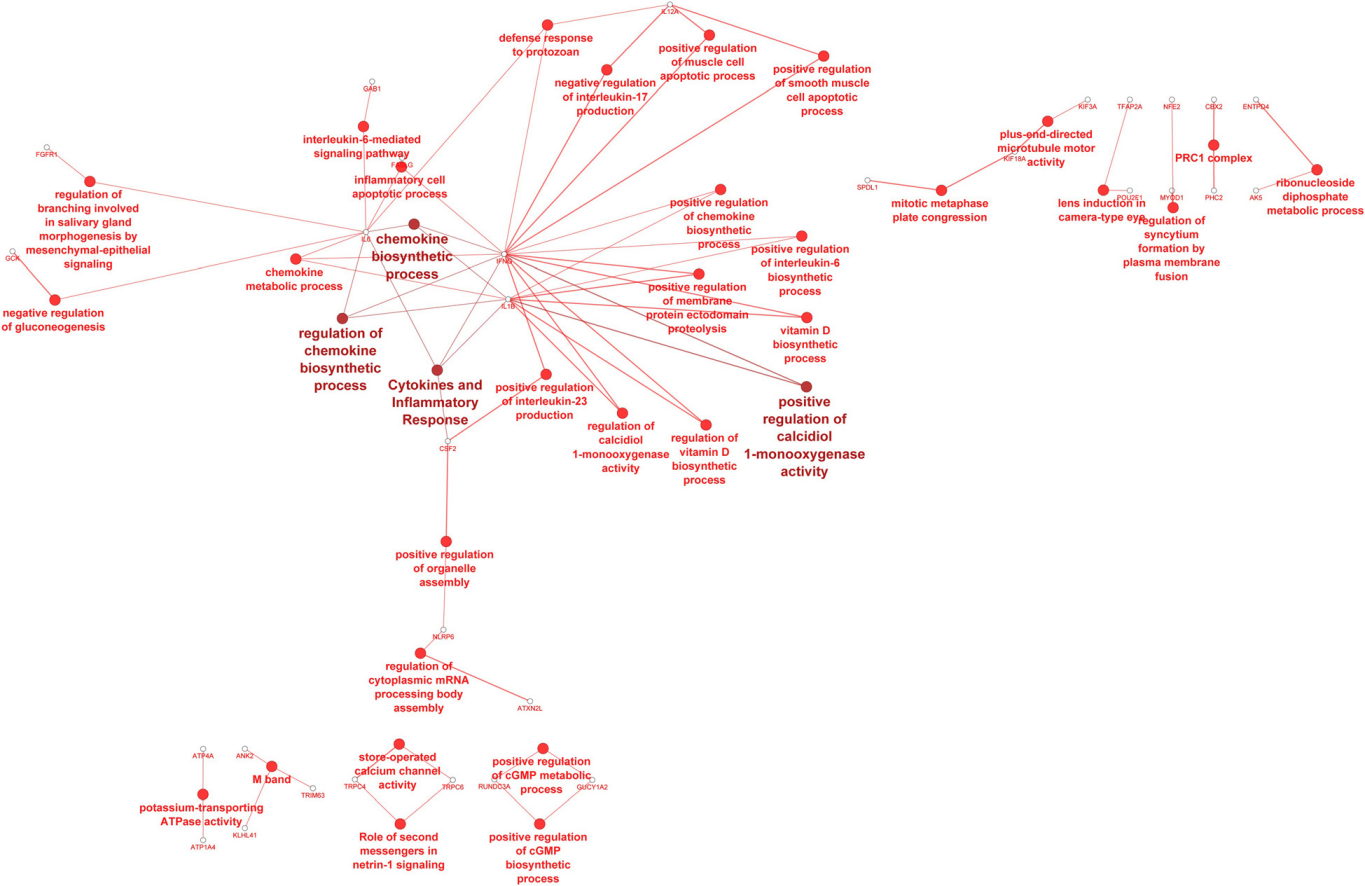
The genes network and biological processes for genes specific to control (C) group. Analysis performed in Cytoscape-ClueGo. The selection criteria for this network were to have at least two genes per node with 15 % of associated genes from all loaded genes in one node. The kappa score threshold was 0.51

Cytoscape classification of DEGs specific for the C group was conducted on 31 genes (Additional file 7). Adjusted pathways included chemokine biosynthetic process, inflammatory response, and regulation of calcidiol 1-monooxygenase activity involved in vitamin D biosynthesis (Fig. 7). Besides the main network, other functions located outside of this network included potassium-transporting ATPase activity, cGMP biosynthetic processes, and syncytium formation.

#### Genes exclusively expressed in both groups

**Fig. 8:**
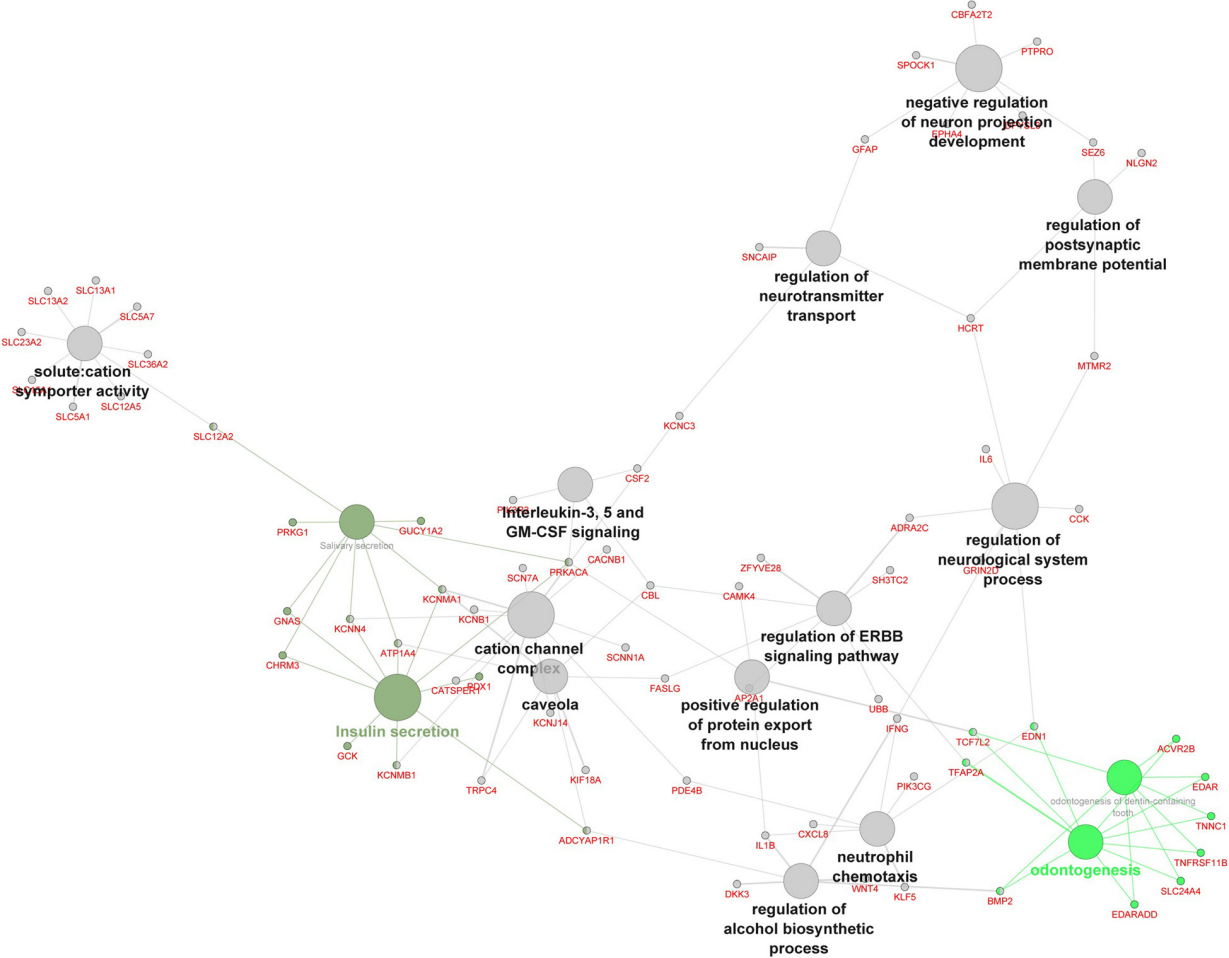
The genes network for genes expressed above detection only in one of the groups merged in one network. Analysis performed in Cytoscape-ClueGo. The data sets of genes expressed only in one of groups were combined together in one analysis to create one figure. The selection criteria for this network were to have at least two genes per node with a minimum of 4 % of the associated genes from all loaded genes in one node. The group percentage was 50 % with a kappa score threshold of 0.4

Cytoscape analysis preformed on DEGs identified exclusively in the E or C group but not in both of the groups showed that the most of the pathways were related to neurological system processes (5 pathways), immunity response (2), and cation transport (2) (Fig. 8). Two specific clusters were assigned in the interaction network processes: regulation of neurological process and regulation of alcohol biosynthetic process.

## Discussion

In our study, the RNA-Seq analysis showed modulatory effects of dietary omega-6 and omega-3 fatty acids on mRNA expression in pig liver. Liver transcriptomes of pigs fed a diet with increased LA and ALA content (the precursors for the synthesis of long-chain PUFA) were compared to those of pigs fed the standard diet. The scope of our study was to investigate the general biological mechanisms underlying the physiological effects of increased dietary intake of PUFA.

### Increase of omega-3 PUFA contribution in fatty acid profile

As we expected, our data indicated specific differences between dietary groups in regard to the fatty acid profile. Results showed a higher contribution of PUFAs among all analyzed series of fatty acids in liver for pigs fed the LA and ALA-enriched diet compared to the standard diet, particularly for the omega-3 fatty acids EPA and DHA, but not omega-6. These results are consistent with those reported for previous gene expression studies in pigs (Corominas et al. 2013) and also rats (Hanke et al. 2013). This finding indicates higher absorption, transport and storage, or lower degradation of unsaturated fatty acids, which suggests inhibition of lipogenesis, but not for the omega-3 pathway. Substantial differences in the omega-6/omega-3 ratio in the liver, lowered ratio in the tissue after PUFA supplementation, outlined that the diet presumably affected body composition, which was the basis for further investigation on the transcriptome level. We can suppose that in the group characterized by increased content of LA and ALA in the diet, appeared specifically increased absorption of omega-3 fatty acids, not omega-6, on the base of fatty acid profile. Even though we consider that the conversion of ALA to EPA and DHA in vivo is less than 2 % (Brenna et al. 2009; Burdge and Calder 2005), the effect on omega-3 pathway was clear.

### Enhancement of fatty acid β-oxidation in liver through induction of the PPAR signaling pathway

Among the most highly DEGs (FC ≥ 8), we identified genes involved in lipid metabolism, which play a key role in the synthesis of fatty acids. We observed lower expression of those genes in the liver of pigs with increased omega-3 fatty acids contribution in the liver, suggesting a major down-regulation of lipid metabolism and increase of β-oxidation. This is thought to relate to the omega-3 and omega-6 fatty acids (Jump 2011). One of the identified genes characterized by significantly reduced expression in our study was CYP7A1, which was shown to be important for the PPAR signaling pathway and is one of the target genes of LXR. The liver X receptor plays a major role in the PPAR signaling pathway and is a key regulator of fatty acid synthesis by regulation of lipogenic gene transcription and induction of SREBP-1c gene expression (Afman and Muller 2012). The effects of omega-3 fatty acids on changes in the expression of LXR’s target genes agreed with the findings of a recent study in pigs (Ramayo-Caldas et al. 2012).

Additionally, the expression of another transcription factor, PPARα, was affected. The peroxisome proliferator-activated receptor is a liver-specific gene and plays a central role in fatty acid β-oxidation in hepatocytes. It is thought to be the main indicator of the fatty acid oxidation-metabolic state (Gormaz et al. 2010). A higher content of omega-6 and omega-3 fatty acids in our study resulted in higher expression of the PPARα gene in the liver. Ramayo-Caldas et al. (2012) reported a similar effect on nuclear factors where the influence on the liver transcriptome of a PUFA-rich diet was compared to monounsaturated fatty acid (MUFA)- and saturated fatty acid (SFA)-enriched diet. Here, we pointed out how different contents of omega-6 and omega-3 PUFAs in the diet resulted in a different ratio of these two fatty acid families in the liver and how these alterations are reflected in the transcriptome. In our study, it is proposed that an omega-6 and omega-3 PUFA-enriched diet induced β-oxidation on the transcriptomic level in comparison to the influence of standard diet. It is generally accepted that omega-3 fatty acids induce hepatic fatty acid oxidation, possibly via PPARα activation, and suppress hepatic fatty acid synthesis through SREBP-1c (for reviews see El-Badry et al. (2007). However, some investigators have reported contrary results. Hanke et al. (2013) showed that omega-3-rich diets had no apparent effect on molecular markers of fatty acid oxidation on protein level. In particular, PPAR and ACOX were not affected nor were markers of fatty acid synthesis (SREBP-1c and ACC1) for livers of rats with diet-induced obesity.

Lastly, we found that up-regulated genes of the genes with the highest value of absolute expression registered by RNA-Seq analysis were involved in fatty acid activation, fatty acid β-oxidation, lipid binding, and lipid transport activity. To summarize, the direction of the expression level for major transcription factors responsible for fatty acid homeostasis, such as PPARα, LXR, or SREBP-1c, showed that omega-6 and omega-3-enriched diets influenced transcription of fatty acid β-oxidation genes and had significant impact on their molecular status what highlight their role as bioactive components of a diet. The higher PPARα expression in the liver suggested the biological modulatory effect of the LA- and ALA-enriched diet at the molecular level.

### Increase of conversion intensity for omega-3 PUFA pathways in the liver

In our study, the FADS2 and FADS1 genes, which are responsible for coding enzymes delta-6-desaturase and delta-5-desaturase, respectively, where up-regulated in the RNA-Seq analysis (validated by qRT-PCR, statistically significant for FADS1). They are common for the conversion of fatty acids on three different pathways of omega fatty acid metabolism, being involved in introducing additional double bonds to dietary PUFAs, linoleic (omega-6) and α-linolenic acid (omega-3), and also to oleic acid (omega-9 MUFA) derived from de novo synthesis or the diet (Ratnayake and Galli (2009).

On the base of transcriptomic analysis, we cannot unambiguously confirm to which pathway delta-6-desaturase and delta-5-desaturase are directed. However, we can hypothesize a potential mechanism from the contributions of the derivatives of omega-6 and omega-3 fatty acid precursors in the fatty acid profile in the liver. Increased content of EPA and DHA (omega-3) and decreased AA (omega-6) suggest that ALA is converted into EPA and further to DHA, but conversion of LA to AA seems to be suppressed. Therefore, we suggest an increased intensity of conversion for omega-3 PUFA in the liver and suppose that FADS2 and FADS1 act mainly in the omega-3 metabolic pathway.

Furthermore, we observed a decreased expression of lipogenic gene coding for acetyl-CoA carboxylase-1 (ACACA). This suggests that the synthesis of short-chain fatty acids was decreased as a consequence of influence of the diet on these lipogenic genes. However, one of another lipogenic gene fatty acid synthase (FASN) was not confirmed by qRT-PCR. Conversely, enhanced activity of the conversion occurs to the long-chain fatty acids related to increased expression of ELOVL6 gene coding elongase (confirmed by qRT-PCR), which convert SFA-palmitic acid to stearic acid and is limiting enzyme for conversion between SFA and MUFA.

The production of acetyl-CoA derived from fatty acid degradation processes does not impact the Krebs cycle independently from the PUFAs content in the diet.

The main difference between the dietary groups in our studies was increased omega-3 intake from the diet and consequently higher conversion of omega-3 in the fatty acid pathway metabolism. Increased contents of omega-6 and omega-3 fatty acids in the diet resulted in altered metabolic pathways in the liver in a specific manner. The main biochemical pathways of unsaturated fatty acid metabolism seem to be directed to the omega-3 pathway, but not omega-6.

Further considerations in our future studies will concern identification of genes participating in the regulation of enzymes involved in lipid metabolism, which are common for different pathways of fatty acid metabolism.

### Activation of pathways related to signal transduction, membrane composition, and mitigation of inflammation state

Analysis in DAVID demonstrated that most of the differentially expressed genes identified among two treatment groups affected by increased omega-3 fatty acid conversion were involved in signal transduction and plasma membrane-related proteins (Table 5). The biological function of signaling pathways is to forward information from the extracellular region via receptors on the plasma membrane and ion channels to support their transfer to the cytoplasm, what is supposed to be influenced by a low ratio of omega-6/omega-3 PUFA in our study and highlighted by the DAVID analysis. For example, extracellular fibronectins, one of the functional clusters revealed by DAVID, are ligands for the integrins located on the plasma membrane surface (Huveneers et al. 2008). An interaction network of reactions mediate between the transmission of various extracellular signals to the nucleus and is responsible for positive regulation of molecular function. All biological functions listed in the functional annotation clustering from DAVID are related to the processes taking place between cell membrane and the extracellular matrix such as ion transport or kinases activation. Polyunsaturated fatty acids are important components of the cell membranes and influence membrane fluidity as well as the action of membrane-bound enzymes and receptors. Thus, it is not surprising that oral administration of omega-6 and omega-3 fatty acids in pigs resulted in alterations emphasizing major functions of fatty acids in organisms—signal transduction and building of membrane, revealed in DAVID and Cytoscape-ClueGo analysis.

DAVID analysis concatenated with the KEGG database further support findings mentioned above and revealed focal adhesion molecules as a significant pathway, which is responsible for signal transduction, functionality of cell membranes, kinases activity, and ion transport. This pathway was related to the PUFA supplemented group, for which the omega-3 fatty acid content was increased.

Additionally, processes specific to pigs fed the supplemental diet (E group) were analyzed, and we observed results consistent with our findings mentioned above concerning the directions of alterations in lipid metabolism. We observed that the regulation of steroid hormone biosynthesis was the most affected pathway of the up-regulated genes, which were expressed only in the group characterized by increased intensity of conversions on omega-3 pathway. Steroid hormones are breakdown products of the EPA and DHA conversion pathways, and these metabolites were influenced by the administration of excess omega-3 fatty acid precursors in the diet.

Conversely, down-regulated genes, expressed only in the standard diet group, represent signaling molecules assigned as a cytokine-cytokine receptor interaction pathway identified by the KEGG database in the DAVID, (see also below) what reflects the anti-inflammatory properties of a high content of omega-3 fatty acids or low ratio of omega-6/omega-3 fatty acids in the liver. The expression of pro-inflammatory cytokines, which occurs in the control group of pigs under a normal physiological condition, is not observed in the PUFA supplemented group, what can hint at mitigation of the inflammation state. Our findings are consistent with studies on omega-3 fatty acid interventions against inflammatory status (Liu et al. 2015). Omega-3 fatty acids have been shown to inhibit expression of cytokine mRNAs, which are stimulated via omega-6 fatty acids (Kruger et al. 2010; Roy et al. 2007). Omega-3 fatty acids disturb the action of omega-6 fatty acid excess. Thus, our results suggest that the diet enriched with omega-3 fatty acids can modulate the immune response and could possibly improve the health of animals.

When we analyzed the processes specific to pigs fed the standard diet, we also observed perturbations of health-related pathways. Under a normal, physiological state, in the standard diet group of pigs, cytokine interaction was the most prominent pathway for genes expressed above detection level registered only in this group, while in the group with increased conversion of omega-3 fatty acids, expression of these genes were either not expressed or were down-regulated. Therefore, we can assume that increased conversion of omega-3 fatty acids revealed anti-inflammatory effect. This is in agreement with a recent study that investigated the effects of different omega-6/omega-3 ratios in pigs (Duan et al. 2014). Duan et al. (2014) revealed that inhibition of immune stimulation in pigs was characterized by a low ratio of omega-6/omega-3 fatty acids. Similarly, Papadopoulos et al. (2009) observed inflammation modulation in sows after administration of high doses of omega-3 fatty acids during the perinatal period. The up-regulation of TNFα in our study, which is an indicator of the inflammatory state, and additionally KEGG pathway and Cytoscape results further support this observation.

Additionally, calcidiol 1-monooxygenase activity, which is involved in vitamin D synthesis, was down-regulated. Vitamin D is known to promote vascular regeneration and possesses anti-proliferative and pro-apoptotic functions. Deficiency of vitamin D was associated with the inflammatory state (Wong et al. 2014). Presence of pro-inflammatory cytokines (such as TNFα and IL-6) might impair activation of vitamin D, which plays a pivotal role in the regulation of the prostaglandin pathway, thereby affecting both PGE2 synthesis by inhibition of COX2 expression (down-regulated in our studies), as well as its degradation by stimulation of 15-PGDH expression (Hummel et al. 2014). Thus, it could be proposed that a higher conversion of omega-3 fatty acids in the liver, caused by increased dietary intake of LA resulted in inhibition of inflammation in vascular tissue possibly via activation of the vitamin D system.

In summary, the presented work utilized high-throughput sequencing to identify the biological and metabolic processes of the pig liver transcriptome most affected by different omega-6 and omega-3 fatty acid content in the diet. A low ratio of omega-6/omega-3 fatty acids in the liver, caused by increased intake of omega-3 fatty acids from the diet, modulated gene expression and affected a set of genes related mainly to energy metabolism, signaling pathways, and the inflammatory response.

## Additional files

Additional file 1:
**The list of genes related to fatty acid metabolism from Cytoscape-ClueGo.** (XLS 409 kb) Additional file 2:
**The list of significantly differentially expressed genes (DEGs).** Significant annotation clusters were listed based on the analysis of functional ontologies using the DAVID tools including all DEGs. (XLS 4445 kb) Additional file 3:
**Cytoscape-ClueGo analysis including all DEGs.** (XLS 212 kb) Additional file 4:
**The list of DEGs specific to experimental (E) group.** A list of significant annotation clusters based on the analysis of functional ontologies using the DAVID tools including DEGs above detection level only in the E diet group. (XLS 1109 kb) Additional file 5:
**Cytoscape-ClueGo analysis, including genes with detectable gene expression found only in the E diet group.** (XLS 423 kb) Additional file 6:
**The list of DEGs specific to control (C) group.** A list of significant annotation clusters based on the analysis of functional ontologies using the DAVID tools including DEGs detected only in the C diet group. (XLS 773 kb) Additional file 7:
**Cytoscape-ClueGo analysis of genes with detectable gene expression found only in the C diet group.** (XLS 162 kb)

## References

[CR1] Afman LA, Muller M (2012). Human nutrigenomics of gene regulation by dietary fatty acids. Prog Lipid Res.

[CR2] Amminger GP, Schafer MR, Schlogelhofer M, Klier CM, McGorry PD (2015). Longer-term outcome in the prevention of psychotic disorders by the Vienna omega-3 study. Nat Commun.

[CR3] Anders S, Huber W (2010). Differential expression analysis for sequence count data. Genome Biol.

[CR4] Aro A (2003). Fatty acid composition of serum lipids: is this marker of fat intake still relevant for identifying metabolic and cardiovascular disorders?. Nutr Metab Cardiovasc Dis.

[CR5] Barrett T, Wilhite SE, Ledoux P, Evangelista C, Kim IF, Tomashevsky M, Marshall KA, Phillippy KH, Sherman PM, Holko M, Yefanov A, Lee H, Zhang N, Robertson CL, Serova N, Davis S, Soboleva A (2013). NCBI GEO: archive for functional genomics data sets—update. Nucleic Acids Res.

[CR6] Bergen WG, Mersmann HJ (2005). Comparative aspects of lipid metabolism: impact on contemporary research and use of animal models. J Nutr.

[CR7] Brazma A, Hingamp P, Quackenbush J, Sherlock G, Spellman P, Stoeckert C, Aach J, Ansorge W, Ball CA, Causton HC, Gaasterland T, Glenisson P, Holstege FCP, Kim IF, Markowitz V, Matese JC, Parkinson H, Robinson A, Sarkans U, Schulze-Kremer S, Stewart J, Taylor R, Vilo J, Vingron M (2001). Minimum information about a microarray experiment (MIAME)—toward standards for microarray data. Nat Genet.

[CR8] Brenna JT, Salem N, Sinclair AJ, Cunnane SC, International Society for the Study of Fatty A, Lipids I (2009). alpha-Linolenic acid supplementation and conversion to n-3 long-chain polyunsaturated fatty acids in humans. Prostaglandins Leukot Essent Fatty Acids.

[CR9] Burdge GC, Calder PC (2005). Conversion of alpha-linolenic acid to longer-chain polyunsaturated fatty acids in human adults. Reprod Nutr Dev.

[CR10] Bustin SA, Benes V, Garson J, Hellemans J, Huggett J, Kubista M, Mueller R, Nolan T, Pfaffl MW, Shipley G, Wittwer CT, Schjerling P, Day PJ, Abreu M, Aguado B, Beaulieu JF, Beckers A, Bogaert S, Browne JA, Carrasco-Ramiro F, Ceelen L, Ciborowski K, Cornillie P, Coulon S, Cuypers A, De Brouwer S, De Ceuninck L, De Craene J, De Naeyer H, De Spiegelaere W, Deckers K, Dheedene A, Durinck K, Ferreira-Teixeira M, Fieuw A, Gallup JM, Gonzalo-Flores S, Goossens K, Heindryckx F, Herring E, Hoenicka H, Icardi L, Jaggi R, Javad F, Karampelias M, Kibenge F, Kibenge M, Kumps C, Lambertz I, Lammens T, Markey A, Messiaen P, Mets E, Morais S, Mudarra-Rubio A, Nakiwala J, Nelis H, Olsvik PA, Perez-Novo C, Plusquin M, Remans T, Rihani A, Rodrigues-Santos P, Rondou P, Sanders R, Schmidt-Bleek K, Skovgaard K, Smeets K, Tabera L, Toegel S, Van Acker T, Van den Broeck W, Van der Meulen J, Van Gele M, Van Peer G, Van Poucke M, Van Roy N, Vergult S, Wauman J, Tshuikina-Wiklander M, Willems E, Zaccara S, Zeka F, Vandesompele J (2013). The need for transparency and good practices in the qPCR literature. Nat Methods.

[CR11] Chen C, Ai H, Ren J, Li W, Li P, Qiao R, Ouyang J, Yang M, Ma J, Huang L (2011). A global view of porcine transcriptome in three tissues from a full-sib pair with extreme phenotypes in growth and fat deposition by paired-end RNA sequencing. BMC Genomics.

[CR12] Chomczynski P, Sacchi N (1987). Single-step method of RNA isolation by acid guanidinium thiocyanate-phenol-chloroform extraction. Anal Biochem.

[CR13] Chomczynski P, Sacchi N (2006). The single-step method of RNA isolation by acid guanidinium thiocyanate–phenol–chloroform extraction: twenty-something years on. Nat Protoc.

[CR14] Corominas J, Ramayo-Caldas Y, Puig-Oliveras A, Estelle J, Castello A, Alves E, Pena RN, Ballester M, Folch JM (2013). Analysis of porcine adipose tissue transcriptome reveals differences in de novo fatty acid synthesis in pigs with divergent muscle fatty acid composition. BMC Genomics.

[CR15] de Pablo MA, Alvarez de Cienfuegos G (2000). Modulatory effects of dietary lipids on immune system functions. Immunol Cell Biol.

[CR16] Duan Y, Li F, Li L, Fan J, Sun X, Yin Y (2014). n-6:n-3 PUFA ratio is involved in regulating lipid metabolism and inflammation in pigs. Br J Nutr.

[CR17] El-Badry AM, Graf R, Clavien PA (2007). Omega 3 - omega 6: what is right for the liver?. J Hepatol.

[CR18] Folch J, Lees M, Sloane Stanley GH (1957). A simple method for the isolation and purification of total lipides from animal tissues. J Biol Chem.

[CR19] Getek M, Czech N, Fizia K, Bialek-Dratwa A, Muc-Wierzgon M, Kokot T, Nowakowska-Zajdel E (2013). Nutrigenomics—bioactive dietary components. Postepy Hig Med Dosw.

[CR20] Gormaz JG, Rodrigo R, Videla LA, Beems M (2010). Biosynthesis and bioavailability of long-chain polyunsaturated fatty acids in non-alcoholic fatty liver disease. Prog Lipid Res.

[CR21] Guillevic M, Kouba M, Mourot J (2009). Effect of a linseed diet on lipid composition, lipid peroxidation and consumer evaluation of French fresh and cooked pork meats. Meat Sci.

[CR22] Hanke D, Zahradka P, Mohankumar SK, Clark JL, Taylor CG (2013). A diet high in alpha-linolenic acid and monounsaturated fatty acids attenuates hepatic steatosis and alters hepatic phospholipid fatty acid profile in diet-induced obese rats. Prostaglandins Leukot Essent Fatty Acids.

[CR23] Harris WS, Assaad B, Poston WC (2006). Tissue omega-6/omega-3 fatty acid ratio and risk for coronary artery disease. Am J Cardiol.

[CR24] Huang DW, Sherman BT, Lempicki RA (2009a) Bioinformatics enrichment tools: paths toward the comprehensive functional analysis of large gene lists. Nucleic Acids Res 37:1–13 10.1093/nar/gkn923 PMC2615629 19033363

[CR25] Huang DW, Sherman BT, Lempicki RA (2009b) Systematic and integrative analysis of large gene lists using DAVID bioinformatics resources. Nat Protoc 4:44–57 10.1038/nprot.2008.211 19131956

[CR26] Hummel DM, Fetahu IS, Groschel C, Manhardt T, Kallay E (2014). Role of proinflammatory cytokines on expression of vitamin D metabolism and target genes in colon cancer cells. J Steroid Biochem Mol Biol.

[CR27] Huveneers S, Truong H, Fassler R, Sonnenberg A, Danen EH (2008). Binding of soluble fibronectin to integrin alpha5 beta1—link to focal adhesion redistribution and contractile shape. J Cell Sci.

[CR28] Jump DB (2011). Fatty acid regulation of hepatic lipid metabolism. Curr Opin Clin Nutr Metab Care.

[CR29] Kanehisa M, Goto S (2000). KEGG: Kyoto encyclopedia of genes and genomes. Nucleic Acids Res.

[CR30] Kanehisa M, Goto S, Sato Y, Kawashima M, Furumichi M, Tanabe M (2014). Data, information, knowledge and principle: back to metabolism in KEGG. Nucleic Acids Res.

[CR31] Kruger MC, Coetzee M, Haag M, Weiler H (2010). Long-chain polyunsaturated fatty acids: selected mechanisms of action on bone. Prog Lipid Res.

[CR32] Lands B (2012). Consequences of essential fatty acids. Nutrients.

[CR33] Li H, Xi Q, Xiong Y, Cheng X, Qi Q, Yang L, Shu G, Wang S, Wang L, Gao P, Zhu X, Jiang Q, Zhang Y, Yuan L (2011). A comprehensive expression profile of microRNAs in porcine pituitary. PLoS ONE.

[CR34] Lionetti L, Mollica MP, Sica R, Donizzetti I, Gifuni G, Pignalosa A, Cavaliere G, Putti R (2014). Differential effects of high-fish oil and high-lard diets on cells and cytokines involved in the inflammatory process in rat insulin-sensitive tissues. Int J Mol Sci.

[CR35] Liu Y-H, Li X-Y, Chen C-Y, Zhang H-M, Kang JX (2015). Omega-3 fatty acid intervention suppresses lipopolysaccharide-induced inflammation and weight loss in mice. Mar Drugs.

[CR36] Lunney JK (2007). Advances in swine biomedical model genomics. Int J Biol Sci.

[CR37] Morine MJ, Tierney AC, van Ommen B, Daniel H, Toomey S, Gjelstad IM, Gormley IC, Perez-Martinez P, Drevon CA, Lopez-Miranda J, Roche HM (2011). Transcriptomic coordination in the human metabolic network reveals links between n-3 fat intake, adipose tissue gene expression and metabolic health. PLoS Comput Biol.

[CR38] Nguyen P, Leray V, Diez M, Serisier S, Le Bloc'h J, Siliart B, Dumon H (2008). Liver lipid metabolism. J Anim Physiol Anim Nutr (Berl).

[CR39] Ntambi JM (1999). Regulation of stearoyl-CoA desaturase by polyunsaturated fatty acids and cholesterol. J Lipid Res.

[CR40] Nygard AB, Jorgensen CB, Cirera S, Fredholm M (2007). Selection of reference genes for gene expression studies in pig tissues using SYBR green qPCR. BMC Mol Biol.

[CR41] Papadopoulos GA, Maes DG, Van Weyenberg S, van Kempen TA, Buyse J, Janssens GP (2009). Peripartal feeding strategy with different n-6: n-3 ratios in sows: effects on sows' performance, inflammatory and periparturient metabolic parameters. Br J Nutr.

[CR42] Pierzchala M, Pareek CS, Urbanski P, Goluch D, Kamyczek M, Rozycki M, Kuryl J (2011). Selection of reference genes for gene expression studies in porcine hepatic tissue using quantitative real-time polymerase chain reaction. Anim Sci Pap Rep.

[CR43] Polawska E, Horbanczuk JO, Pierzchala M, Strzalkowska N, Jozwik A, Wojcik A, Pomianowski J, Gutkowska K, Wierzbicka A, Hoffman LC (2013). Effect of dietary linseed and rapeseed supplementation on fatty acid profiles in the ostrich. Part 1. Muscles. Anim Sci Pap Rep.

[CR44] Ramakers C, Ruijter JM, Deprez RH, Moorman AF (2003). Assumption-free analysis of quantitative real-time polymerase chain reaction (PCR) data. Neurosci Lett.

[CR45] Ramayo-Caldas Y, Mach N, Esteve-Codina A, Corominas J, Castello A, Ballester M, Estelle J, Ibanez-Escriche N, Fernandez AI, Perez-Enciso M, Folch JM (2012). Liver transcriptome profile in pigs with extreme phenotypes of intramuscular fatty acid composition. BMC Genomics.

[CR46] Ratnayake WM, Galli C (2009) Fat and fatty acid terminology, methods of analysis and fat digestion and metabolism: a background review paper. Ann Nutr Metab 55:8–43 10.1159/000228994 19752534

[CR47] Roy N, Barnett M, Knoch B, Dommels Y, McNabb W (2007). Nutrigenomics applied to an animal model of inflammatory bowel diseases: transcriptomic analysis of the effects of eicosapentaenoic acid- and arachidonic acid-enriched diets. Mutat Res.

[CR48] Ruijter JM, Ramakers C, Hoogaars WM, Karlen Y, Bakker O, van den Hoff MJ, Moorman AF (2009). Amplification efficiency: linking baseline and bias in the analysis of quantitative PCR data. Nucleic Acids Res.

[CR49] Ruijter JM, Pfaffl MW, Zhao S, Spiess AN, Boggy G, Blom J, Rutledge RG, Sisti D, Lievens A, De Preter K, Derveaux S, Hellemans J, Vandesompele J (2013). Evaluation of qPCR curve analysis methods for reliable biomarker discovery: bias, resolution, precision, and implications. Methods.

[CR50] Saito R, Smoot ME, Ono K, Ruscheinski J, Wang PL, Lotia S, Pico AR, Bader GD, Ideker T (2012). A travel guide to Cytoscape plugins. Nat Methods.

[CR51] Shi R, Chiang VL (2005). Facile means for quantifying microRNA expression by real-time PCR. Biotechniques.

[CR52] Stanley WC, Cox JW, Asemu G, O'Connell KA, Dabkowski ER, Xu W, Ribeiro RF, Shekar KC, Hoag SW, Rastogi S, Sabbah HN, Daneault C, des Rosiers C (2013). Evaluation of docosahexaenoic acid in a dog model of hypertension induced left ventricular hypertrophy. J Cardiovasc Transl Res.

[CR53] Vallim T, Salter AM (2010). Regulation of hepatic gene expression by saturated fatty acids. Prostaglandins Leukot Essent Fatty Acids.

[CR54] Warensjo E, Sundstrom J, Lind L, Vessby B (2006). Factor analysis of fatty acids in serum lipids as a measure of dietary fat quality in relation to the metabolic syndrome in men. Am J Clin Nutr.

[CR55] Wong MS, Leisegang MS, Kruse C, Vogel J, Schurmann C, Dehne N, Weigert A, Herrmann E, Brune B, Shah AM, Steinhilber D, Offermanns S, Carmeliet G, Badenhoop K, Schroder K, Brandes RP (2014). Vitamin d promotes vascular regeneration. Circulation.

[CR56] Wymann MP, Schneiter R (2008). Lipid signalling in disease. Nat Rev Mol Cell Biol.

[CR57] Ye J, Coulouris G, Zaretskaya I, Cutcutache I, Rozen S, Madden TL (2012). Primer-BLAST: a tool to design target-specific primers for polymerase chain reaction. BMC bioinformatic.

